# Isolation and Bioactivity of Natural Products from *Streptomyces* sp. MA37

**DOI:** 10.3390/molecules30020306

**Published:** 2025-01-14

**Authors:** Fleurdeliz Maglangit, Qing Fang, Jioji N. Tabudravu, Kwaku Kyeremeh, Marcel Jaspars, Hai Deng

**Affiliations:** 1 Department of Biology and Environmental Science, College of Science, University of the Philippines Cebu, Lahug, Cebu City 6000, Philippines; 2 Marine Biodiscovery Centre, Department of Chemistry, School of Natural and Computing Sciences, University of Aberdeen, Old Aberdeen AB24 3UE, UK; cyanfong1610@gmail.com (Q.F.); m.jaspars@abdn.ac.uk (M.J.); 3 School of Pharmacy and Biomedical Sciences, University of Central Lancashire, Preston PR1 2HE, UK; jtabudravu@uclan.ac.uk; 4 Marine and Plant Research Laboratory of Ghana, Department of Chemistry, University of Ghana, Legon-Accra P.O. Box LG56, Ghana; kkyeremeh@ug.edu.gh

**Keywords:** legonoxamine, hydroxamate, hydroxylamine, iron chelators

## Abstract

The isolation and characterization of bioactive metabolites from *Streptomyces* species continue to represent a vital area of research, given their potential in natural product drug discovery. In this study, we characterize a new siderophore called legonoxamine I, together with a known compound, streptimidone, from the talented soil bacterium *Streptomyces* sp. MA37, using chromatographic techniques and spectroscopic analysis. Legonoxamine I is a new holo-siderophore, which is likely to be a derailed product from the biosynthetic pathway of legonoxamine A. We also demonstrate that legonoxamine A possesses potent anticancer activity (IC_50_ = 2.2 µM), exhibiting a remarkable ~30-fold increase in potency against MCF-7 ATCC HTB-22 breast cancer cells compared to desferrioxamine B, a structural analogue of legonoxamine A (IC_50_ = 61.1 µM). Comparing the structural difference between legonoxamine A and desferrioxamine B, it is deduced that the phenylacetyl moiety in legonoxamine A may have contributed significantly to its enhanced potency. Our findings contribute to the growing library of *Streptomyces*-derived metabolites and underscore the genus’ potential as a promising source of lead compounds.

## 1. Introduction

The genus *Streptomyces* is renowned for its outstanding ability to synthesize a plethora of bioactive molecules, many of which exhibit potent anticancer properties. As a prolific source of natural products, *Streptomyces* species have contributed to the discovery and development of over two-thirds of clinically relevant antibiotics and numerous other therapeutic agents. *Streptomyces’* unique biosynthetic capabilities enable the synthesis of a diverse array of specialized metabolites, which have demonstrated efficacy against multiple cancer cell lines. Hence, exploring *Streptomyces* as a source of novel anticancer compounds is crucial to identifying new lead compounds that could enhance the current treatment strategies while minimizing adverse side effects.

**Figure 1:**
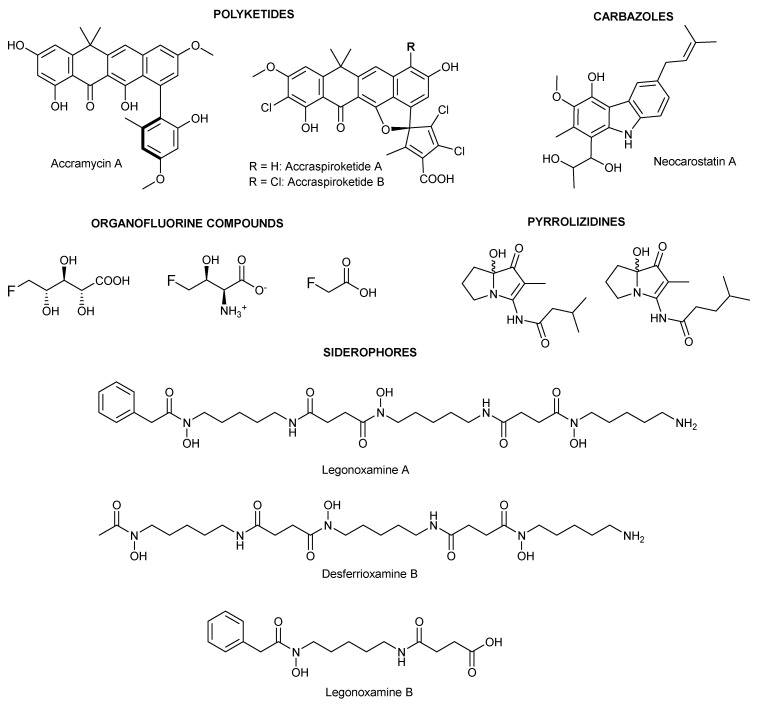
Representative natural products isolated from *Streptomyces* sp. MA37.

The talented soil bacterium *Streptomyces* sp. MA37 has been a prolific source of diverse natural products, including polyketides, spiroketides, siderophores, pyrrolizidines, carbazoles, and organofluorine compounds [1,2] (Figure 1). Continued mining for unique scaffolds from the MA37 strain in our laboratory has led to the isolation of a new siderophore, which is likely a derailed product from the legonoxamine A biosynthetic pathway [3,4].

Siderophores are specialized molecules secreted by microorganisms to scavenge iron from their environment, an essential nutrient that is often limited in availability [5]. These molecules can exist in two primary forms: apo-siderophores (unbound) and holo-siderophores (iron-complexed). Apo-siderophores are iron-free and actively sequester iron ions (typically Fe^3^⁺) from the surroundings. Once iron binds to the apo-siderophore, it becomes a holo form recognized by specific receptors on the microbial cell surface, thereby allowing the transport of the iron complex into the cell. Inside the cell, the iron is released from the siderophore for use in bacterial growth and other essential metabolic processes.

In response to low iron availability, bacteria primarily produce unbound siderophores, making the apo form the most commonly isolated and widely studied [6,7]. Although uncommon, previous studies have reported the isolation of iron-bound siderophores from bacteria such as ferrioxamine G, D, and E from *Hafnia alvei* and *Erwinia amylovora* [8,9]. Herein, we present the structure determination for the new siderophore, legonoxamine I, along with the known glutarimide-containing polyketide, streptimidone, from *Streptomyces* sp. MA37. Additionally, we also report the novel anticancer activity of the legonoxamine A siderophore against breast cancer cells.

## 2. Results and Discussion

The *Streptomyces* sp. MA37 was cultivated under iron-limiting conditions to induce siderophore production. Repeated rounds of chromatographic fractionation led to the isolation of a new siderophore, legonoxamine I, along with the known compound streptimidone. Through advanced spectroscopic methods, including nuclear magnetic resonance (NMR) and mass spectrometric analysis, we elucidated their structures.

### 2.1. Structure Elucidation

**Figure 2:**
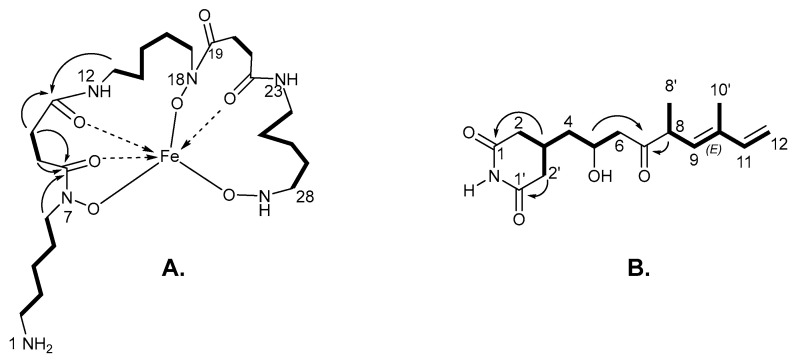
(**A**). COSY (–) and key HMBC (→) correlations of legonoxamine I; (**B**). COSY (–) and key HMBC (→) correlations of streptimidone.

**Figure 3:**
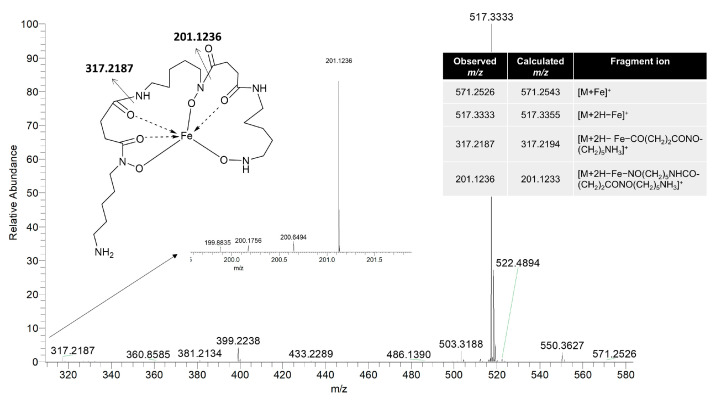
Assignment of fragment ions of the mass spectrum of legonoxamine I.

High-resolution electrospray ionization mass spectrometry (HR ESI MS) provided a molecular ion peak of compound **1** at *m*/*z* 571.2526 [M + Fe]^+^, consistent with the calculated molecular weight for C_23_H_43_FeN_6_O_7_ (calculated [M + Fe]^+^, 571.2543; ∆ = −2.706 ppm), with five degrees of unsaturation (Figure 2A, Figure 3 and Figure S1).

A closer examination of the MS also revealed a dominant ion peak at 517.33 Da, suggesting the presence of the unbound form of the siderophore [M + 2H − Fe]^+^. Fragmentation analysis revealed peaks at 317.21 Da and 201.12 Da (Figure 3), corresponding to breakdown products involving the cleavage between succinyl and hydroxamate bonds, indicating the presence of the typical tri-hydroxamate core characteristic of ferrioxamine-type siderophores [9]. The hydroxamate functional group plays a crucial role in the chelating ability of siderophores, particularly in iron-deficient environments. Composed of an amide (–CONH–) and a hydroxyl group (–OH), the hydroxamate forms a bidentate site capable of forming coordinate bonds with ferric ions (Fe^3^⁺). Multiple hydroxamate groups in a siderophore molecule often coordinate simultaneously, creating a highly stable octahedral complex with iron [10]. This strong stable binding allows siderophores to capture and solubilize iron effectively, making it accessible to microorganisms that require it for critical biological processes such as DNA synthesis, respiration, and enzyme catalysis [3,11]. However, the distinct isotopic pattern due to the natural abundances of iron isotopes, such as ^56^Fe and ^54^Fe, was not observed in the MS spectrum.

A comprehensive examination of the 1D and 2D NMR spectra, including ^1^H, ^13^C, COSY, HSQC, and HMBC experiments (Figures S2–S5), provided evidence for the presence of a siderophore in its iron-free (unbound) state (Table 1). A detailed analysis of the spectral data suggested that compound **1** was closely related to the previously isolated apo-siderophores, legonoxamine A and desferrioxamine B, from *Streptomyces* sp. MA37 (Figure 1) [3,4].

The ^1^H and ^13^C-NMR spectra and HSQC analysis revealed the presence of 23 carbon signals, including 19 methylene carbons and 4 quaternary carbonyls (δ_C_ 174.2, 173.6, 173.4, and 173.4). Analysis of the ^1^H-^1^H COSY spectrum revealed two main spin systems, including three repeating aminopentane motifs (H-2 to H-6, H13 to H-17, and H-24 to H-28) and two recurring succinyl motifs (H-9 to H-10 and H-20 to H-21). The heteronuclear multiple-bond correlations (HMBCs) from H-9 (δ_H_ 2.46) and H-10 (δ_H_ 2.59) to C-8 (δ_C_ 173.4) and C-11 (δ_C_ 173.4), and from H-20 (δ_H_ 2.46) and H-21 (δ_H_ 2.59) to C-19 (δ_C_ 173.6) and C-22 (δ_C_ 174.2), indicated the presence of two succinyl groups. The long-range couplings observed in HMBC experiments established the connectivity of the five main substructures from H-6 (δ_H_ 3.21) to the carbonyl carbon at C-8, H-13 (δ_H_ 3.19) to C-11, H-17 (δ_H_ 3.21) to C-19, and H-24 (δ_H_ 3.19) to C-22.

Two potential non-complexed forms of compound **1** were proposed, with deprotonation at either the N7 or N18 position (Figure S6). To assess the plausibility of each structure, predicted chemical shifts (calculated using the ACD/Structure Elucidator; ACD/Labs 2019.2.0, version S05S41) [12] were compared against experimental chemical shifts obtained in CD_3_OD at 600 MHz. The ACD/Labs Structure Elucidator uses the HOSE algorithm [13] to calculate chemical shifts, which has been known to predict the correct chemical structures of natural products [12,14,15]. Results revealed an excellent linear correlation, with an *R*^2^ value of 0.9991 for the N7-deprotonated form and 0.9995 for the N18-deprotonated form, indicating that both structures closely match the observed data (Figure S6). ACD Labs was unable to distinguish between the two non-complexed structures. Taken together, compound **1**, which we named legonoxamine I, represents a new siderophore, which is likely to be a derailed product from the biosynthetic pathway of legonoxamine A [3,4].

The molecular formula of compound **2**, also isolated as a white powder, was established as C_16_H_22_NO_4_ by HRESIMS (calculated as [M + H]^+^ = 294.1700; observed [M + H]^+^ = 294.1703; ∆ = 1.0198 ppm), indicating six degrees of unsaturation (Figure 2B and Figure S7).

The ^1^H and ^13^C NMR spectra indicated the presence of 22 protons and 16 carbons (2 CH_3_, 5 CH_2_, 5 CH, and 4 C). A comparison of the 1D and 2D NMR data of compound **2** to the known data for glutarimide antibiotics reported in the literature [16,17,18,19] indicated that compound **2** is the known compound, i.e., streptimidone or 4-(2-hydroxy-5,7-dimethyl-4-oxo-6,8-nonadienyl)-2,6-piperidinedione (Table S1; Figures S8–S13).

The double-bond configuration was assigned as *E* based on the absence of a cross-peak between H-9 and the methyl protons at C-10 in the NOESY spectrum, indicating that these protons are spatially distant and positioned on opposite sides of the double bond. Furthermore, the chemical shift values closely match those of known models of *E*-1,3-dienes [18], providing strong evidence for this stereochemical assignment.

This study reaffirms the significance of *Streptomyces* as a source of diverse natural products. The combined production of streptimidone and legonoxamine I by *Streptomyces* sp. MA37 likely represents a competitive strategy that enhances its ecological fitness in resource-limited environments. Glutarimide-containing polyketides such as streptimidone are known for their potent antifungal properties, e.g., the ability to suppress the growth of competing microbes such as *Phytophthora capsici*, *Didymella bryoniae*, *Magnaporthe grisea*, and *Botrytis cinerea* in plants [16]. Furthermore, the natural streptimidone stereoisomer exhibits the most potent antifungal activity, while those lacking the glutarimide moiety are inactive against fungi [17]. Siderophores such as legonoxamine I, on the other hand, help *Streptomyces* sequester iron from the environment, an essential nutrient often limited in availability, while depriving competitors of access to this resource. Together, compounds **1** and **2** ensure that *Streptomyces* thrives by both suppressing competitors and securing essential resources for its growth and metabolism.

### 2.2. Cell Proliferation Assay

Neither legonoxamine A nor desferrioxamine B showed cell proliferation against skin cancer (A2058 ATCC CRL-11147). However, legonoxamine A displayed potent anticancer activity against MCF-7 ATCC HTB-22 breast cancer cells with a half-maximal inhibitory concentration (IC_50_) of 2.2 µM (Table 2). Compared to its structural analogue, desferrioxamine B (IC_50_ = 61.1 µM), legonoxamine A exhibited a remarkable ~30-fold increase in potency. Based on the structural differences between the two siderophores, the phenyl acetyl moiety in legonoxamine A may have contributed significantly to its enhanced cytotoxic activity.

It is also noteworthy that legonoxamine A did not exhibit cytotoxic effects against normal lung cancer cell lines (ATCC CCL-171) at the highest concentration tested (50 µg/mL). The results suggest that legonoxamine A may selectively target breast cancer cells without harming normal healthy cells, which is a desirable property for potential therapeutic agents. Our findings contribute to the growing library of *Streptomyces*-derived metabolites and underscore the genus’s potential as a promising source of bioactive agents.

## 3. Materials and Methods

### 3.1. Reagents and Media

All reagents used in the experiment were obtained from Fisher Scientific (Lancaster, UK). Unless otherwise stated, all media used were sourced from Oxoid (Hampshire, UK).

### 3.2. Extraction and Isolation

The isolation of the producing strain, *Streptomyces* sp. MA37, was described in previous reports [3,20]. The seed culture of MA37 was prepared by inoculating glycerol stock into an iron-limited medium, ISP2 (2.0 g of glucose, 2.0 g of yeast extract, and 5.0 g of malt extract in 500 mL Milli-Q H_2_O) in a 1:10 ratio. The culture was then incubated for 3 days in a rotary shaker maintained at 28 °C and 220 rpm (Incu-shake FL16-2). This seed culture was then used to prepare 10 L of bacterial fermentation, maintaining the same iron-limited broth, inoculation ratio, and fermentation conditions. Subsequently, Diaion^®^ HP-20 (3 g/50 mL) was added to the culture broth and incubated under the same fermentation conditions for 24 h to adsorb secondary metabolites.

The mixture was then filtered under vacuum, and the recovered HP-20 resin was rinsed with Milli-Q water to remove any residual biomass and culture medium. The adsorbed compounds were subsequently eluted from the resin by extracting it with 100% methanol thrice. All the methanol extracts were combined, concentrated under reduced pressure at 40 °C using a rotary evaporator (Buchi Rotavapor R200, Langholm, UK), and subjected to high-resolution electrospray ionization mass spectrometry (HRESIMS) analysis. The crude extract was fractionated on a Strata^®^ C18 SPE column using a solvent system of methanol in water (i.e., 25%, 50%, 75%, and 100% MeOH), representing a gradient of decreasing polarity. Finally, the column was flushed with 100% methanol containing 0.05% trifluoroacetic acid (TFA). This process resulted in the collection of five distinct fractions (F25, F50, F75, F100, and F100TFA). The fractions were then concentrated under reduced pressure, as described above, and subjected to HRESIMS analysis.

Compound **1** was detected in F100, while compound **2** was detected in F100TFA using HRESIMS and ^1^H NMR analysis. Further purification was carried out using high-performance liquid chromatography (HPLC; Agilent 1260 Infinity) with a reversed-phase C18 semi-preparative column (ACE 10 µM 10 × 250 mm). The purification was achieved using a linear gradient from 15% H_2_O:MeOH:TFA (95:5:0.1) to 100% MeOH for 45 min with a solvent flow rate of 1.5 mL/min to yield legonoxamine I (**1**) (3.0 mg) and streptimidone (**2**) (2.0 mg).

### 3.3. NMR and MS Measurements

HR-ESIMS was determined using the LC-MS Thermo Scientific MS system (LTQ Orbitrap, Hemel Hempstead, UK) coupled to a Thermo Instrument HPLC system (Accela PDA detector, Accela PDA autosampler, and Accela pump, C18 Sunfire 150 × 46 mm Waters^®^, UK). The following parameters were used: a capillary voltage of 45 V, a capillary temperature of 320 °C, an auxiliary gas flow rate of 10–20 arbitrary units, a sheath gas flow rate of 40–50 arbitrary units, a spray voltage of 4.5 kV, and a mass range of 100–2000 amu (with a maximum resolution of 30,000). A high-performance digital Bruker AVANCE III HD 600 MHz (Ascend™ 14.1 Tesla, UK) and a Prodigy TCI™ cryoprobe were used to obtain the following information at 25 °C: ^1^H NMR, ^13^C NMR, ^1^H–^1^H COSY, ^1^H–^13^C HSQC,^1^H–^13^C HMBC, and NOESY.

### 3.4. Cell Proliferation Assay

The antiproliferative activity of siderophores was tested against skin cancer (A2058 ATCC CRL-11147) and breast cancer cells (MCF-7 ATCC HTB-22 using the CellTiter 96^®^ Aqueous One Solution Cell Proliferation Assay, following the protocol described previously [21]. Meanwhile, the toxicity was tested on the lung normal cells (ATCC CCL-171). The cell lines were seeded in 96-well-microtitre plates (Nunc, Thermo Fisher Scientific, New York, NY, USA) at 2000 cells/well in Dulbecco’s Modified Eagle Medium (DMEM, Thermo Fisher Scientific, Waltham, MA, USA) with fetal bovine serum (10%, FBS, Sigma Aldrich, St. Louis, MO, USA) and gentamicin (10 µg/mL, Sigma Aldrich, USA). Cells were incubated at 37 °C in a 5% CO_2_ atmosphere for 24 h. The wells were then treated with various concentrations of the pure compounds to a total volume of 100 μL/well. The plate was then incubated for 72 h at 37 °C and 5% CO_2_. After treatment, 10 µL of CellTiter 96^®^ Aqueous One Solution Reagent (Promega, Madison, WI, USA) was added to each well, and the cells were incubated for 1–4 h at 37 °C. Cell viability was determined based on the conversion of 3-(4,5-dimethylthiazol-2-yl)-5-(3-carboxymethoxyphenyl)-2-(4-sulfophenyl)-2H-tetrazolium (MTS) tetrazolium (yellow) to a formazan product (blue) by metabolically active cells. The absorbance was measured at 485 nm using a microplate reader (Multimode detector DTX 880). The quantity of formazan product, as measured by the absorbance, is directly proportional to the number of living cells in the culture. The dose-dependent response of the pure compounds (100 ng/mL, 1, 2.5, 5, 10, 12.5, 25, and 50 µg/mL) was tested in triplicates. Cell viability was expressed as mean ± standard deviation.

## 4. Conclusions

The successful isolation of both legonoxamine I siderophore and streptimidone polyketide from *Streptomyces* sp. MA37 species underscores its remarkable biosynthetic potential. Our findings also highlight the potential of legonoxamine A to inhibit breast cancer cell proliferation selectively, warranting further investigation into its mechanisms of action. *Streptomyces*’s unique biosynthetic capabilities provide an opportunity to discover diverse metabolites that may exhibit selective toxicity toward cancer cells while minimizing harm to normal tissues. The genus *Streptomyces* continues to be a valuable resource for natural product discovery.

## Figures and Tables

**Table 1:** ^1^H and ^13^C NMR data of Compound **1** at 600 MHz and 298 K in CD_3_OD.

No.	13C ppm *	^1^H ppm, mult.	COSY	HMBC
1-N	-	-	-	-
2	39.0, CH_2_	2.92, tr	3, 4, 5, 6	3, 4
3	28.3, CH_2_	1.54, m	2, 4, 5, 6	2, 4
4	22.8, CH_2_	1.39, m	2, 3, 5, 6	3, 5, 6
5	25.9, CH_2_	1.68, m	2, 3, 4, 6	2, 3, 4, 6
6	50.5, CH_2_	3.21, m	2, 3, 4, 5	4, 8, 9
7-NOH	-	-	-	
8	173.4, C	-	-	
9	29.8, CH_2_	2.46, m	10	8, 9, 11
10	28.3, CH_2_	2.59, m	9	8, 10, 11
11	173.4, C	-	-	-
12-NH	-	-	-	-
13	39.4, CH_2_	3.19, m	14, 15, 16, 17	14, 15
14	28.3, CH_2_	1.54, m	13, 15, 16, 17	13, 15
15	22.8, CH_2_	1.39, m	13, 14, 16, 17	14, 16, 17
16	25.9, CH_2_	1.68, m	13, 14, 15, 17	13, 14, 15, 17
17	50.5, CH_2_	3.21, m	13, 14, 15, 16	15, 19, 20
18-NOH	-	-	-	-
19	173.6, C	-	-	-
20	29.8, CH_2_	2.46, m	21	19, 21, 22
21	28.3, CH_2_	2.59, m	20	19, 20, 22
22	174.2, C	-	-	-
23-NH	-	-	-	-
24	39.4, CH_2_	3.19, m	25, 26, 27, 28	25, 26
25	28.3, CH_2_	1.54, m	24, 26, 27, 28	24, 26
26	22.8, CH_2_	1.39, m	24, 25, 27, 28	24, 27, 28
27	28.3, CH_2_	1.54, m	24, 25, 26, 28	24, 25, 26, 28
28	46.8, CH_2_	3.63, t	24, 25, 26, 27	26, 27
29-NO	-	-	-	

* deduced from HMBC and HSQC data.

**Table 2:** Cytotoxic activity of legonoxamine A and desferrioxamine B against skin cancer, breast cancer, and lung normal cell lines.

Compound Name	IC_50_		
	A2058 ATCC CRL-11147 (*Skin Cancer*)	MCF-7 ATCC HTB-22 (*Breast Cancer*)	ATCC CCL-171 (*Lung Normal Cell*)
Legonoxamine A	-	2.2 µM	-
Desferrioxamine B	-	61.1 µM	-

- indicates no activity at the highest concentration tested (50 µg/mL).

## Data Availability

Data are contained within the article and Supplementary Materials.

## Supplementary Materials

The following supporting information can be downloaded at: https://www.mdpi.com/article/10.3390/molecules30020306/s1, Figure S1. HRESIMS of legonoxamine I; Figure S2. ^1^H-NMR of unbound legonoxamine I (CD_3_OD, 600 MHz, 298 K); Figure S3. HSQC of unbound legonoxamine I (CD_3_OD, 600 MHz, 298 K); Figure S4. ^1^H-^1^H COSY of unbound legonoxamine I (CD_3_OD, 600 MHz, 298 K); Figure S5. HMBC of unbound legonoxamine I (CD_3_OD, 600 MHz, 298 K); Figure S6. Experimental vs. predicted ^13^C chemical shifts for unbound legonoxamine I (1) with A. deprotonation at N7 (resulting in the formation of NO^−^) and B. deprotonation at N18; Figure S7. HRESIMS of streptimidone; Figure S8. ^1^H-NMR of streptimidone (CD_3_OD, 600 MHz, 298 K); Figure S9. ^13^C-NMR of streptimidone (CD_3_OD, 600 MHz, 298 K); Figure S10. ^1^H-^1^H COSY of streptimidone (CD_3_OD, 600 MHz, 298 K); Figure S11. HSQC of streptimidone (CD_3_OD, 600 MHz, 298 K); Figure S12. HMBC of streptimidone (CD_3_OD, 600 MHz, 298 K); Figure S13. NOESY of streptimidone (CD_3_OD, 600 MHz, 298 K); Table S1. Comparison of the ^1^H and ^13^C Chemical Shifts of compound 2 with those of streptimidone reported in the literature.
