# Supplementary material for: Isolation and Bioactivity of Natural Products from Streptomyces sp. MA37
Source: Molecules. 2025 Jan 14;30(2):306. doi: 10.3390/molecules30020306 (PMC11767966; doi:10.3390/molecules30020306)
Supplement: Supplementary file 1 [file molecules-30-00306-s001.zip › molecules-3365639-supplementary.pdf]

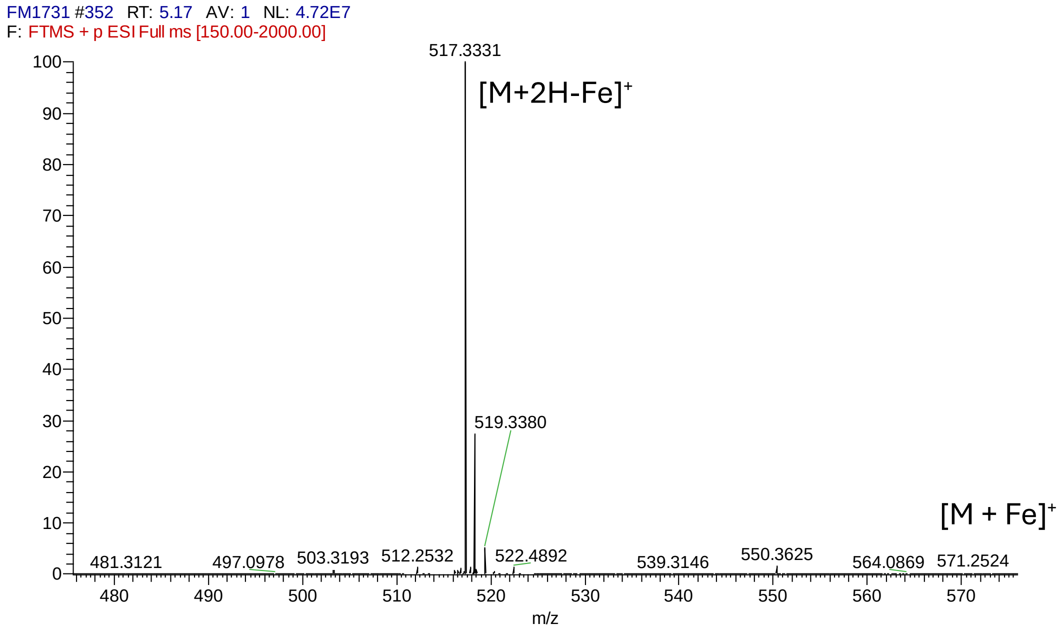

Figure S1. HRESIMS of legonoxamine I

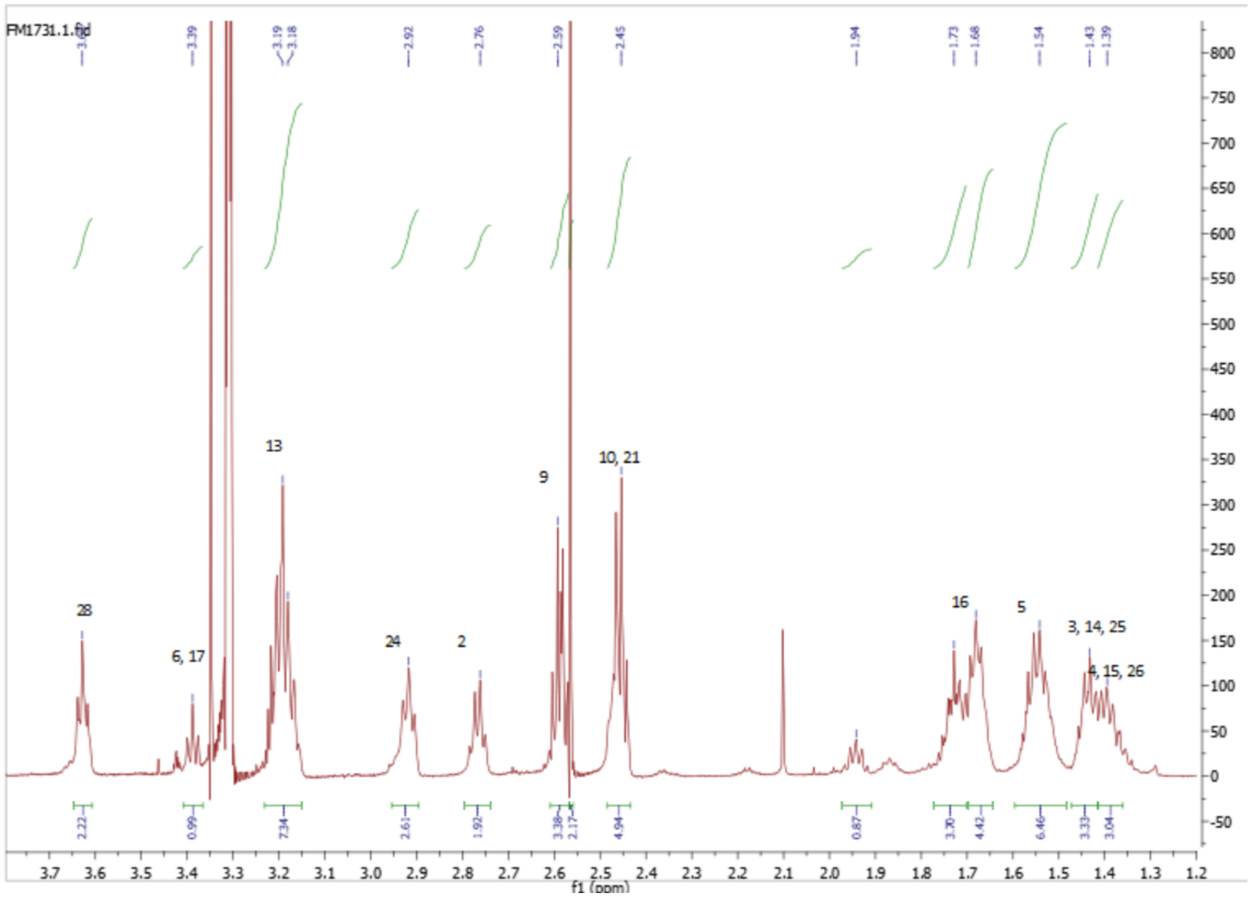

Figure S2. ^{1}H-NMR of unbound legonoxamine I (CD_3OD, 600 MHz, 298 K)

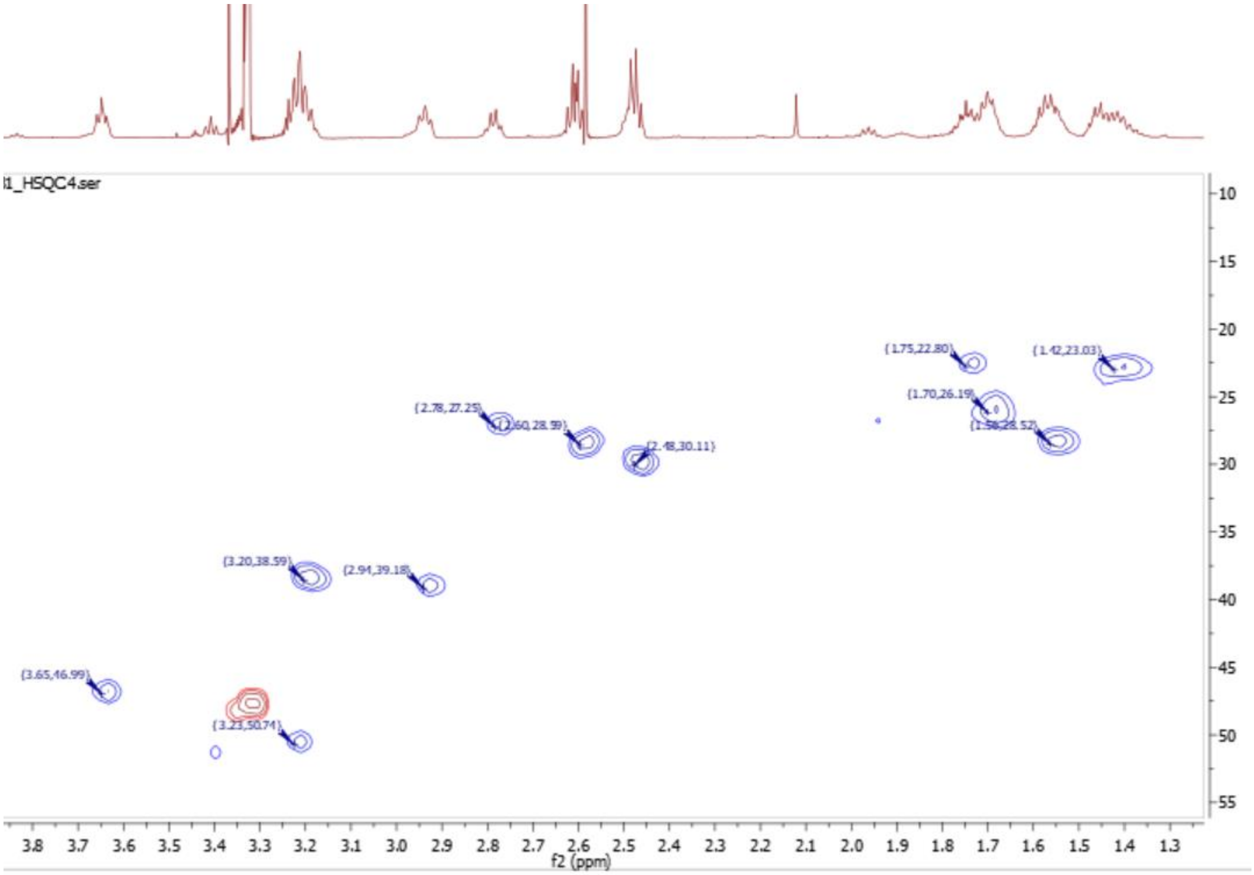

Figure S3. HSQC of unbound legonoxamine I (CD_3OD, 600 MHz, 298 K)

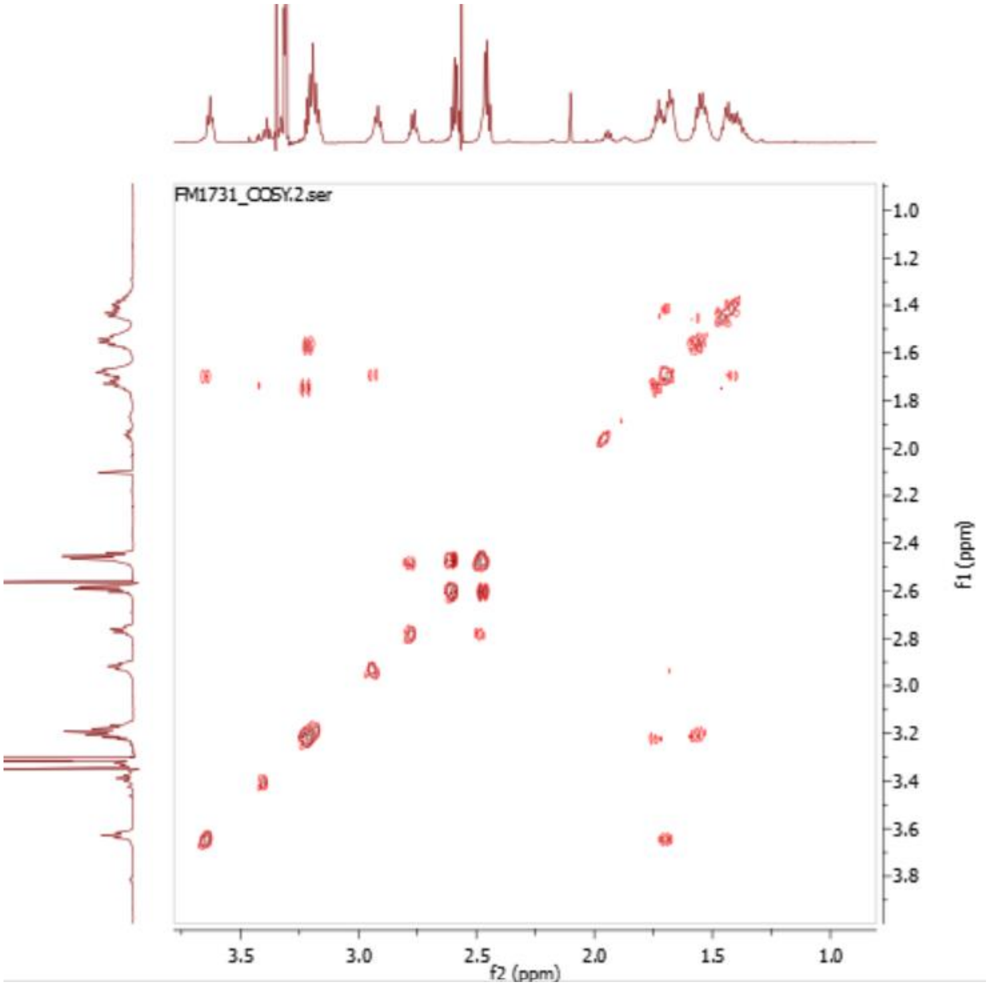

Figure S4. ^{1}H-^{1}H COSY of unbound legonoxamine I (CD_3OD, 600 MHz, 298 K)

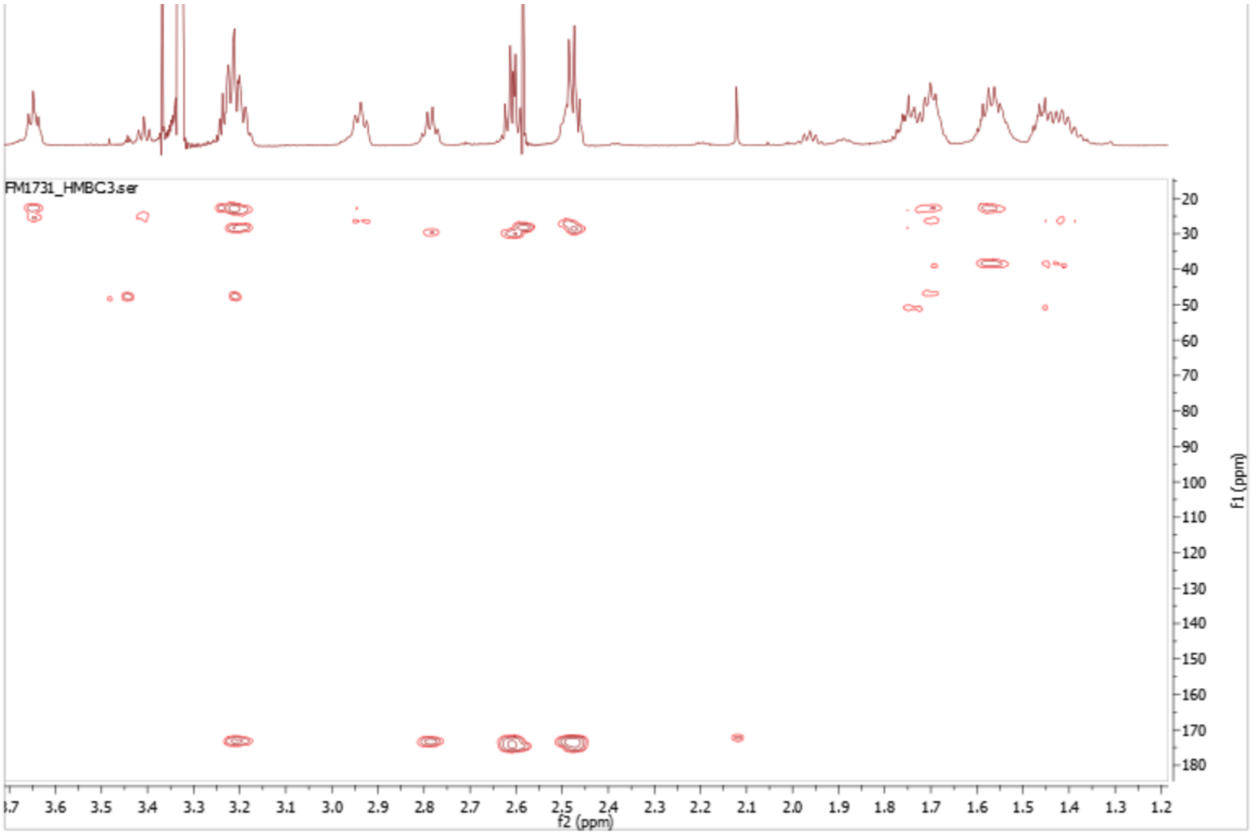

Figure S5. HMBC of unbound legonoxamine I (CD_3OD, 600 MHz, 298 K)

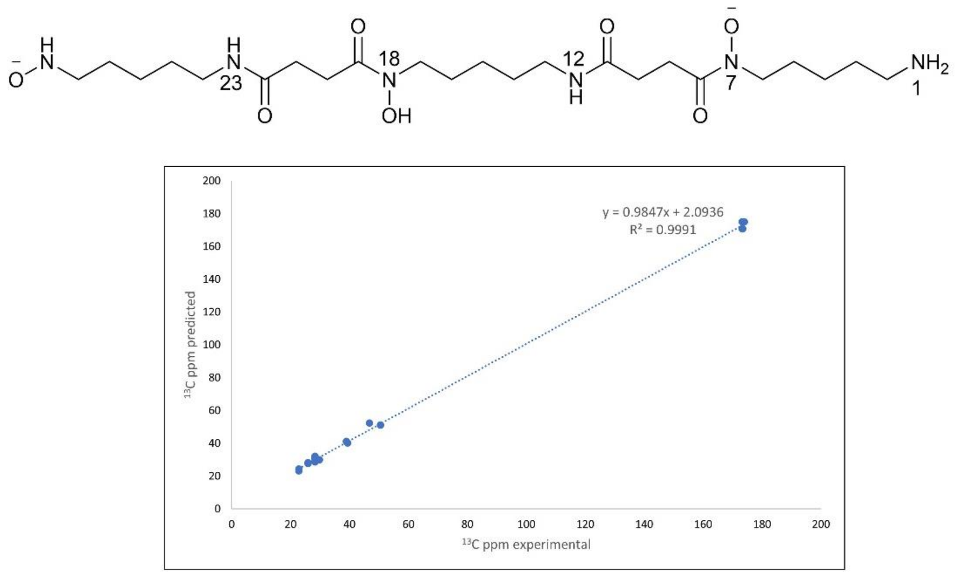

A

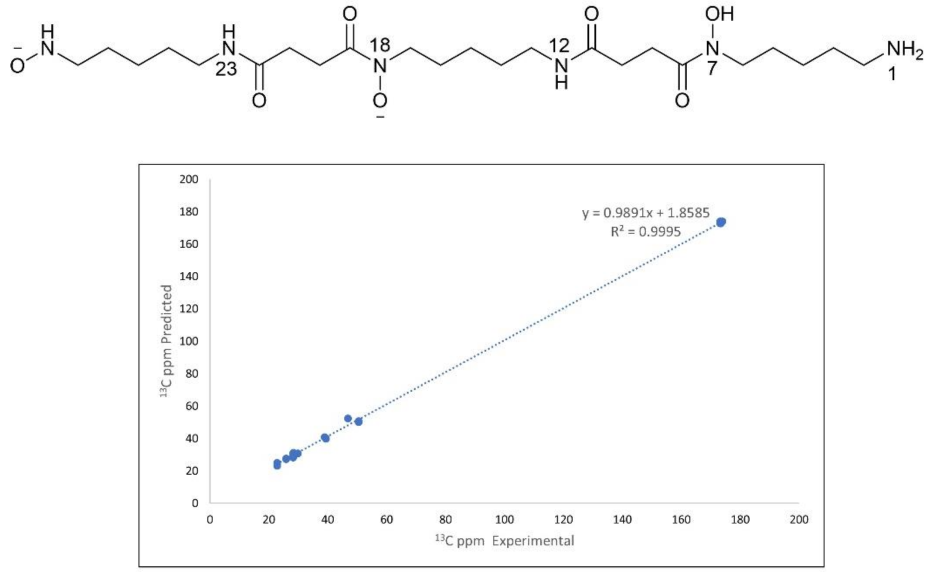

B

Figure S6. Experimental vs. predicted ^{13}C chemical shifts for unbound legonoxamine I (**1**) with **A**. deprotonation at N7 (resulting in the formation of NO^-) and **B**. deprotonation at N18.

Table S1. Comparison of the ^{1}H and ^{13}C Chemical Shifts of compound **2** with those of streptimidone reported in the literature

No.	Compound **2**		Reported [16−19]	
	δ_H (ppm), mult. (*J* in Hz)	δ_C (ppm), mult.	δ_H (ppm), mult. (*J* in Hz)	δ_C (ppm), mult.
1	-	174.27, C	-	172.6, C
1’	-	174.27, C	-	172.7, C
2	2.66, m<br>2.32, m	37.72, CH_2	a: 2.75, m<br>b: 2.27, m	37.0, CH_2
2’	2.66, m<br>2.32, m	38.21, CH_2	a: 2.72, m<br>b: 2.30, m	38.2, CH_2
3	2.32, m	28.31, CH	2.45, m	27.0, CH
4	a: 1.50, ddd (14.1, 10.4, 5.0)<br>b: 1.15, ddd (14.1, 8.9, 2.8)	42.60, CH_2	a: 1.57, ddd (14.1, 10.4, 5.0)<br>b: 1.30, ddd (14.0, 8.7, 3.0)	40.8, CH_2
5	4.13, m	65.45, CH	4.09, m	64.6, CH
6	a: 2.66, m (18.0, 8.5)<br>b: 2.66, dd (18.0, 2.9)	47.62, CH_2	a: 2.59, dd (17.9, 8.4)<br>b: 2.53, dd (17.9, 3.3)	47.5, CH_2
7	-	210.33, C	-	211.9, C
8	3.62, m	47.62, CH	3.48, m	46.8, CH
8Me	1.15, d (6.7)	16.14, CH_3	1.15, d (6.8)	16.0, CH_3
9	5.34, d (9.7)	130.91, CH	5.31, d (9.7)	130.1, CH
10	-	136.20, C	-	136.5, C
10Me	1.85, s (1.24)	12.04, CH_3	1.80, d (1.24)	12.1, CH_3
11	6.38, dd	140.42, CH	6.33, dd (17.4, 10.7)	140.4, CH
12	a: 5.19, dt (17.4, 10.7)<br>b: 5.05, d (10.7)	112.69, CH_2	a: 5.18, d (17.4, 10.7)<br>b: 5.03, d (10.7)	112.9, CH_2
NH	-		8.49, brs	

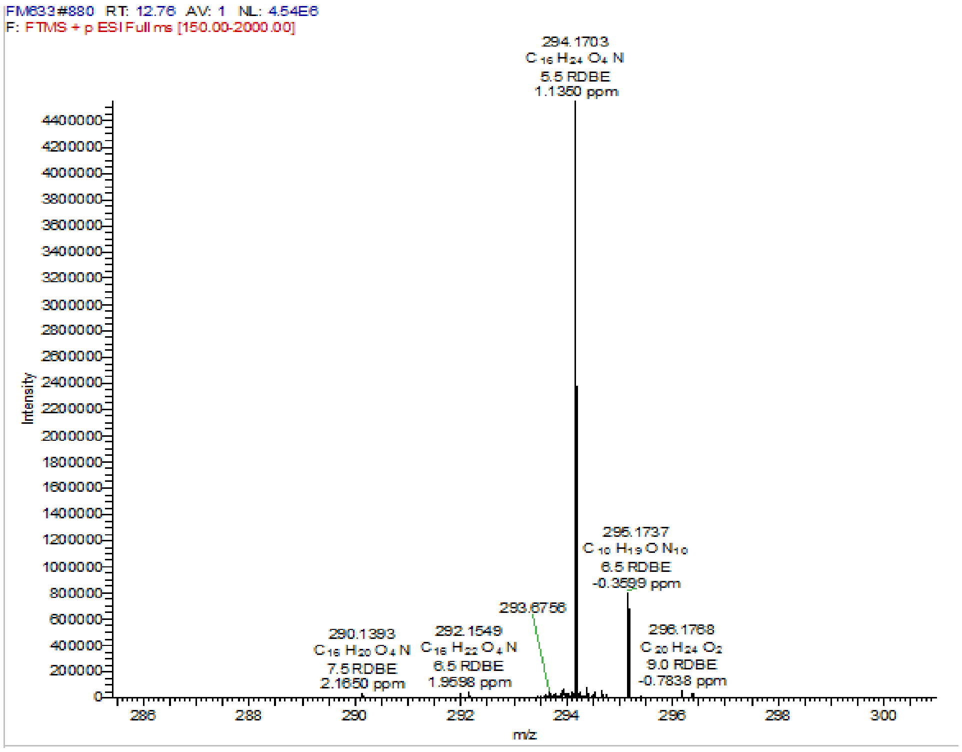

Figure S7. HRESIMS of streptimidone

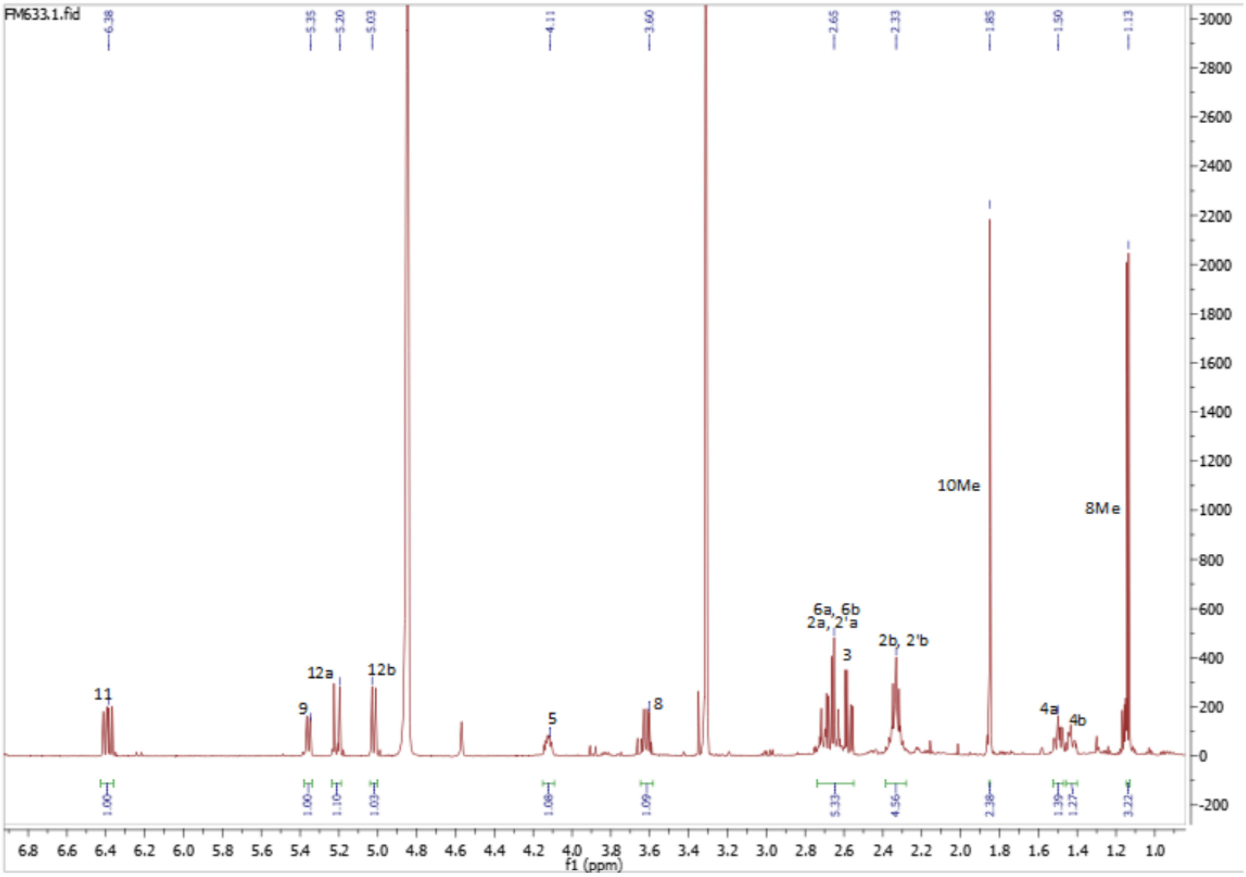

Figure S8. ^{1}H-NMR of streptimidone (CD_3OD, 600 MHz, 298 K)

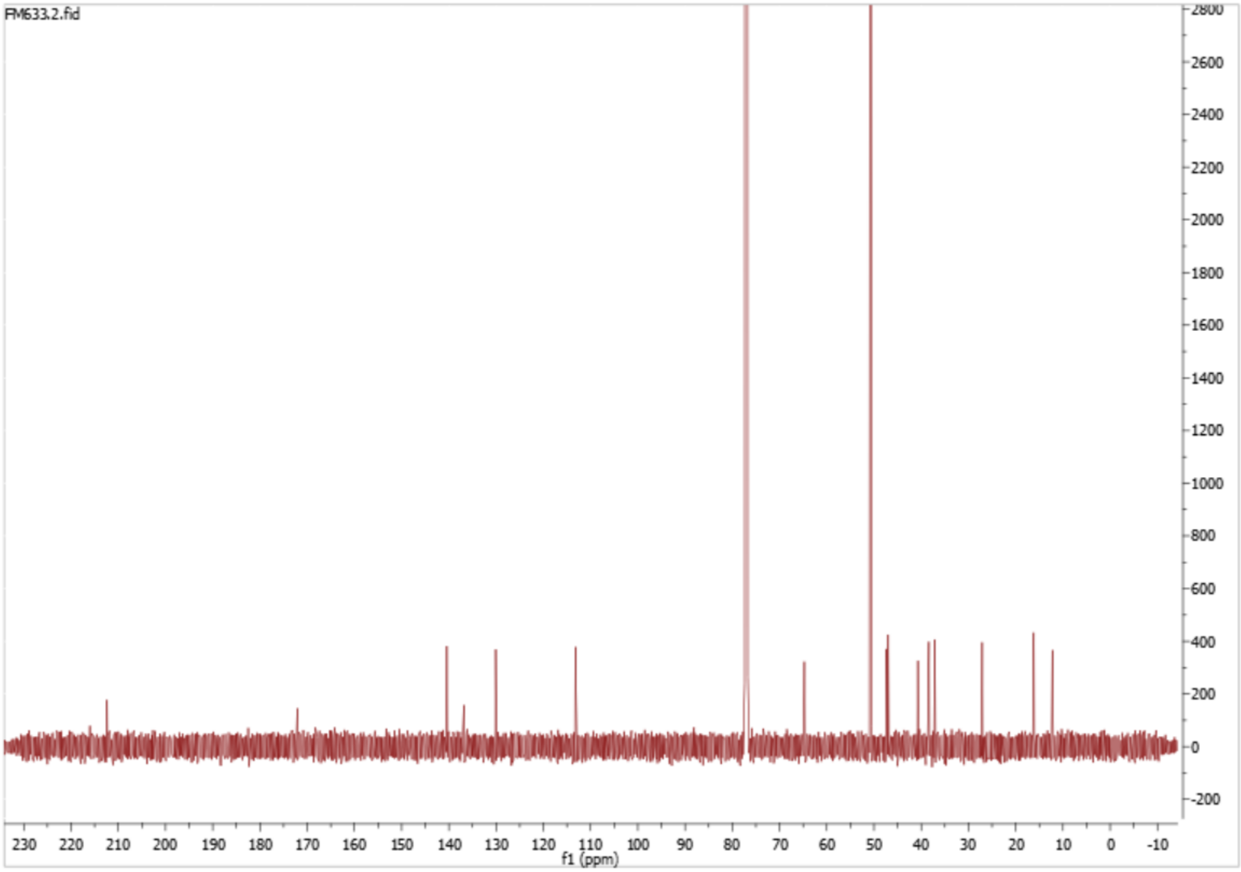

Figure S9. ^{13}C-NMR of streptimidone (CD_3OD, 600 MHz, 298 K)

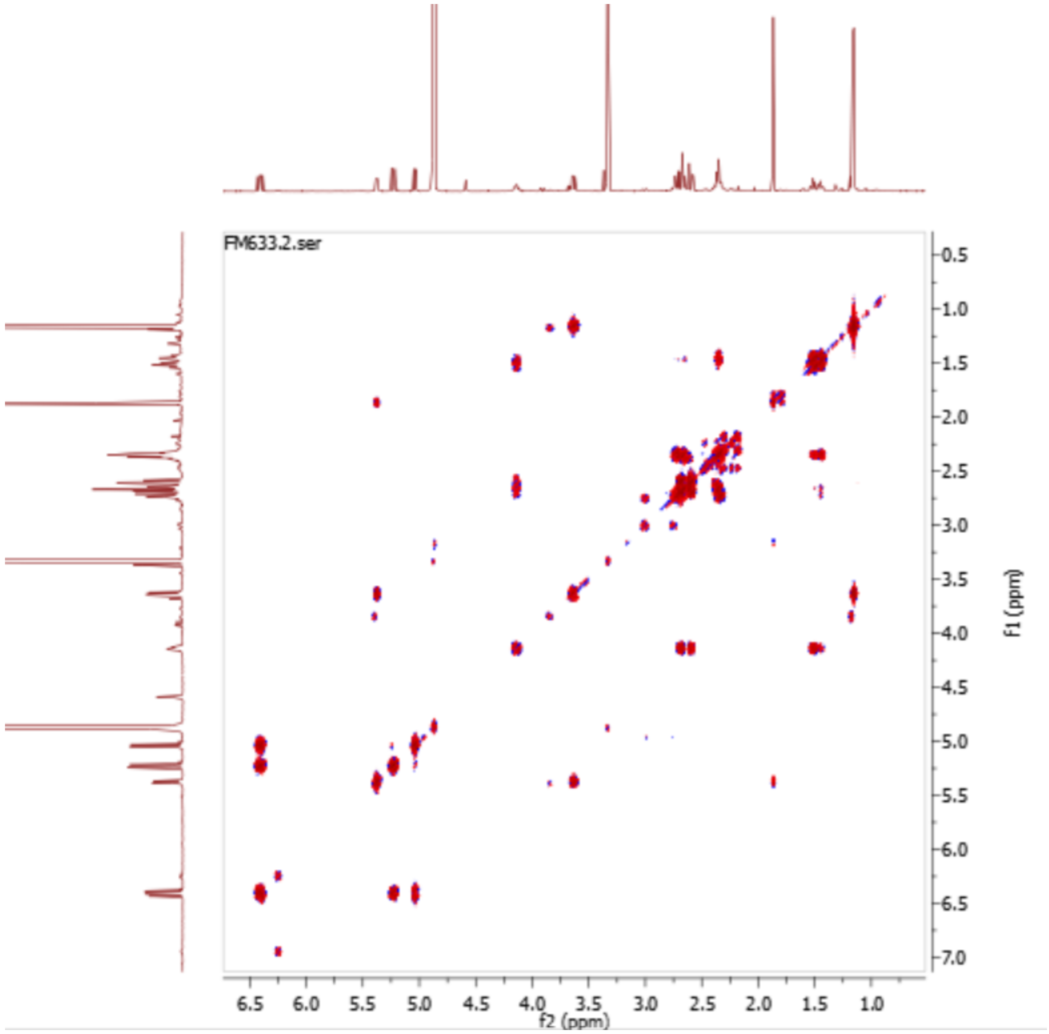

Figure S10. ^{1}H-^{1}H COSY of streptimidone (CD_3OD, 600 MHz, 298 K)

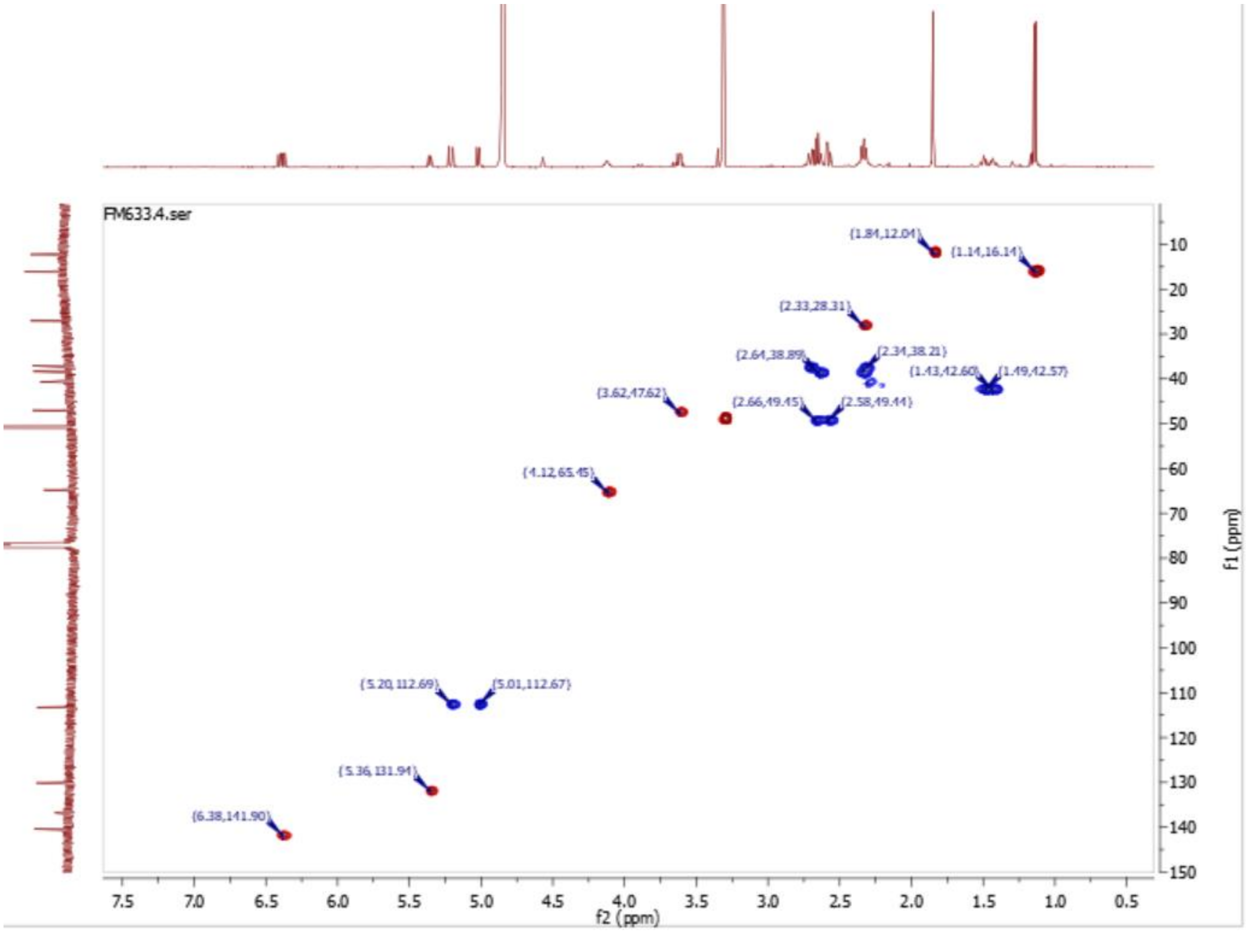

Figure S11. HSQC of streptimidone (CD_3OD, 600 MHz, 298 K)

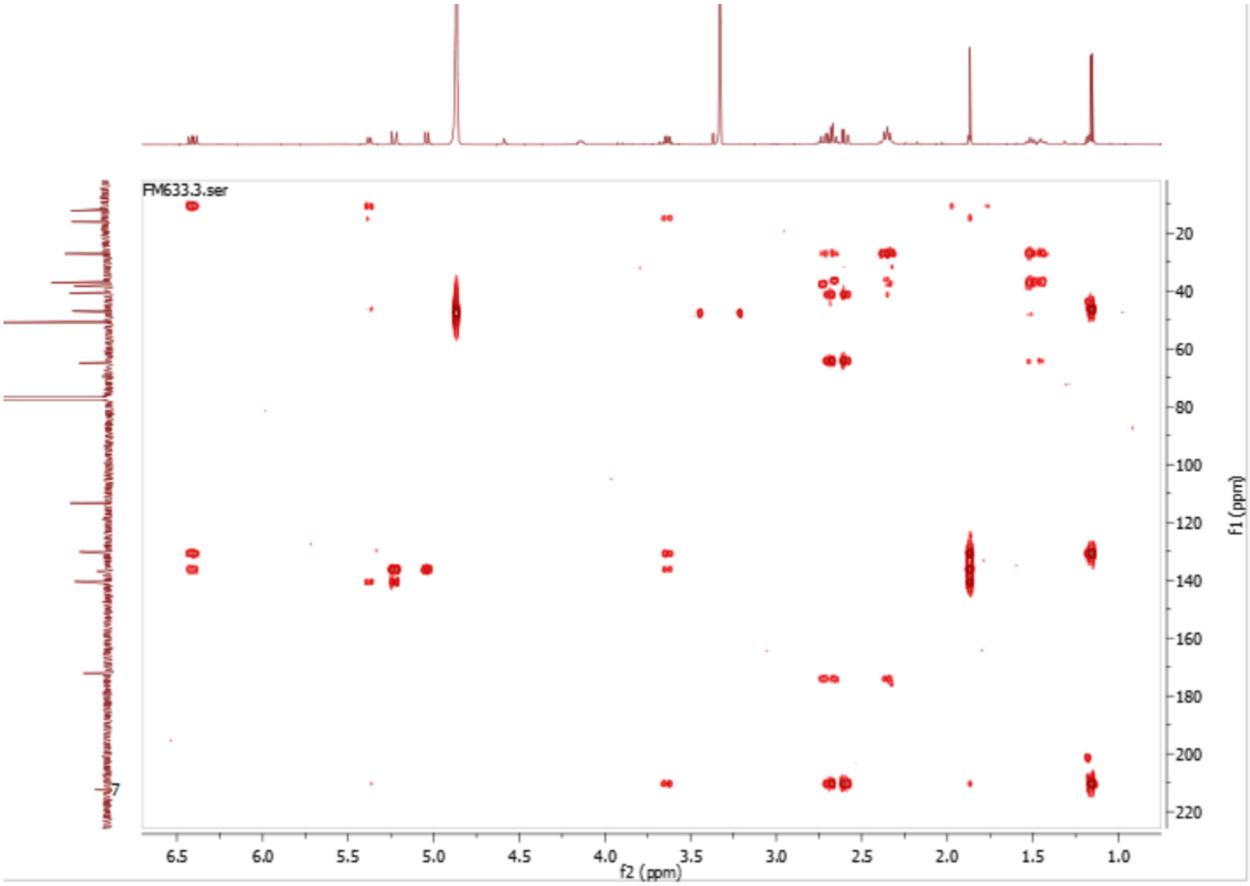

Figure S12. HMBC of streptimidone (CD_3OD, 600 MHz, 298 K)

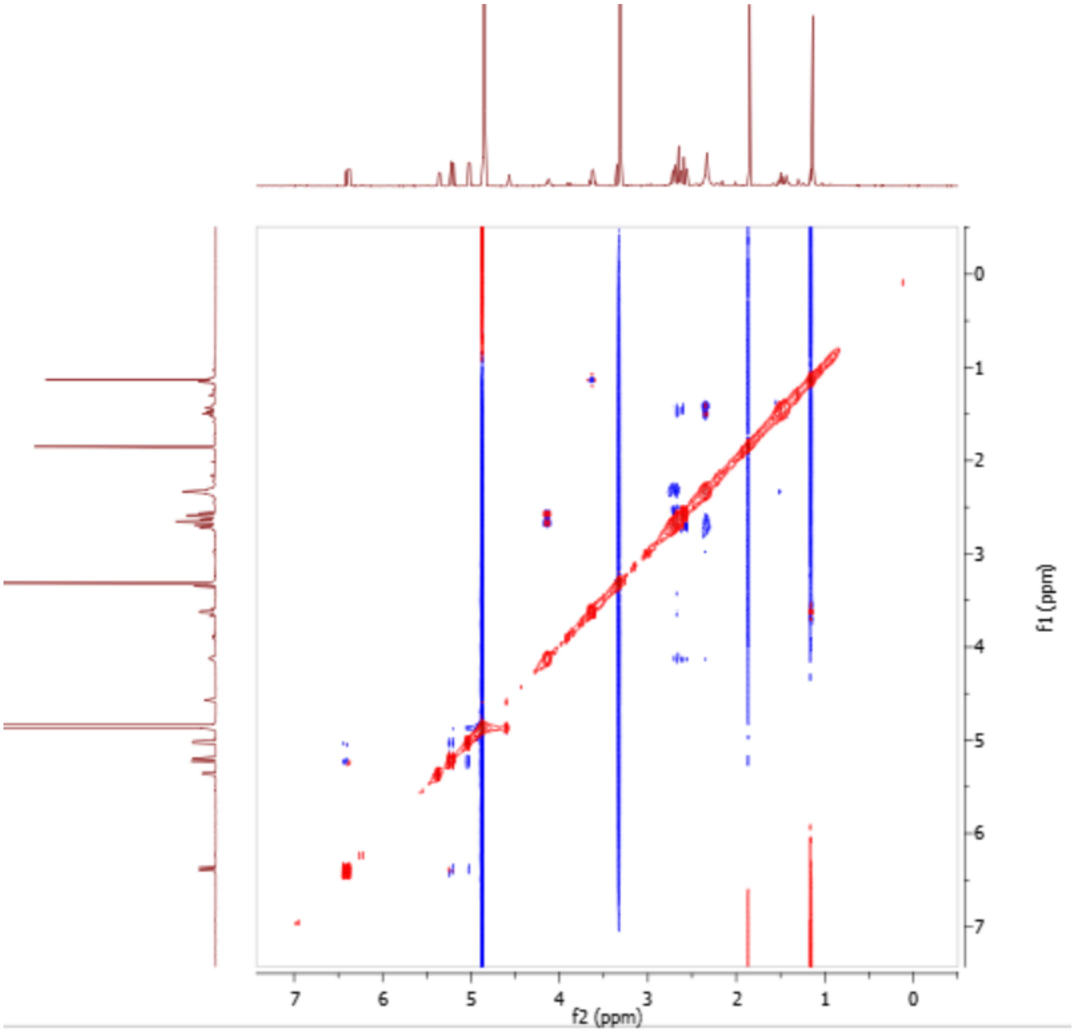

Figure S13. NOESY of streptimidone (CD_3OD, 600 MHz, 298 K)
